# Desmopressin nasal spray inhibiting parasympathetic function on isolated tracheal smooth muscle

**DOI:** 10.7150/ijms.98166

**Published:** 2024-07-08

**Authors:** Ying-Liang Chou, Hsing-Won Wang

**Affiliations:** 1 Department of Otolaryngology-Head and Neck Surgery, Taichung Armed Forces General Hospital, Taichung, Taiwan, Republic of China.; 2 Department of Otolaryngology-Head and Neck Surgery, Tri-Service General Hospital, National Defense Medical Center, Taipei, Taiwan, Republic of China.; 3 Department of Medical Imaging and Radiological Sciences, Central Taiwan University of Science and Technology, Taichung, Taiwan, Republic of China.; 4 The Graduate Institute of Clinical Medicine and Department of Otolaryngology, College of Medicine, Taipei Medical University-Shuang Ho Hospital, Taipei, Taiwan, Republic of China.

**Keywords:** Desmopressin, trachea, smooth muscle, *in vitro* study

## Abstract

***Objectives:*** Nocturia with or without asthma is one of the aging diseases. Desmopressin has been used as a nasal spray for patients who are suffering from nocturia. This study determined the effects of desmopressin on isolated tracheal smooth muscle *in vitro*.

***Methods:*** We evaluated desmopressin's efficiency on isolated rat tracheal smooth muscle. Desmopressin was evaluated for the following effects on tracheal smooth muscle: (1) effect on resting tension; (2) effect on contraction brought on by parasympathetic mimetic 10^-6^ M methacholine; and (3) effect on electrically produced tracheal smooth muscle contractions.

***Results:*** As the concentration grew, desmopressin by itself had no impact on the trachea's baseline tension. Addition of desmopressin at doses of 10^-5^ M or above elicited a significant relaxation response to 10^-6^ M methacholine-induced contraction. Desmopressin could also inhibit spike contraction of the trachea induced by electrical field.

***Conclusion:*** According to this study, desmopressin at high quantities may prevent the trachea's parasympathetic activity. Due to its ability to block parasympathetic activity and lessen the contraction of the tracheal smooth muscle brought on by methacholine, Desmopressin nasal spray might help nocturia sufferers experience fewer asthma attacks.

## Introduction

Nocturia is the bothersome condition of waking two or more times per night to pass urine. Desmopressin (deamino-8-D-arginine vasopressin) is a drug that regulates urine volume and concentration in the body. It is also used as a nasal spray to treat nocturia. It is made from a synthetic analogue of the human hormone vasopressin, which has been used for decades. The posterior pituitary gland produces vasopressin, which maintains serum osmolality and volume by modulating free water excretion. Hyperosmolality and hypovolemia are detected by chemoreceptors and baroreceptors located in the hypothalamus and carotid sinus, respectively. Vasopressin is released as an antidiuretic hormone. It also has a minor role in increasing systemic vascular resistance and increasing urea reabsorption in the medullary collecting tubule. Desmopressin has traditionally been used to treat central diabetes insipidus, bleeding disorders such as Von Willebrand disease, and primary nocturnal enuresis 1,2. The US Food and Drug Administration approved desmopressin nasal spray for nocturia due to nocturnal polyuria in adults who awaken at least twice per night to void. The adverse events for desmopressin include headache, hypertension, hyponatremia, insomnia, dry mouth, abdominal pain, peripheral edema, and nausea. Difficulty in breathing and chest tightness were also noted. However, the effects of desmopressin on the trachea have not been explored 3-5.

Most studies on desmopressin have focused on nocturnal polyuria and bleeding disorders 4-7. Nocturia or asthma is one of aging-associated diseases. Urine storage symptoms and voiding symptoms were proved significantly higher in the asthma group than in the non-asthma group 8. Because nocturia patients may have comorbid asthma, it is reasonable to explore the role of desmopressin nasal spray in tracheal smooth muscle. During an asthmatic attack, the tracheal smooth muscle may reduce pulmonary function as it becomes contracted. This study used rat tracheas to apply a simple *in vitro* technique to test the effects of drugs that induce tracheal constriction or relaxation. The aim of this study was to determine the effects of desmopressin on isolated tracheal smooth muscle *in vitro*.

## Materials and methods

**Figure 1:**
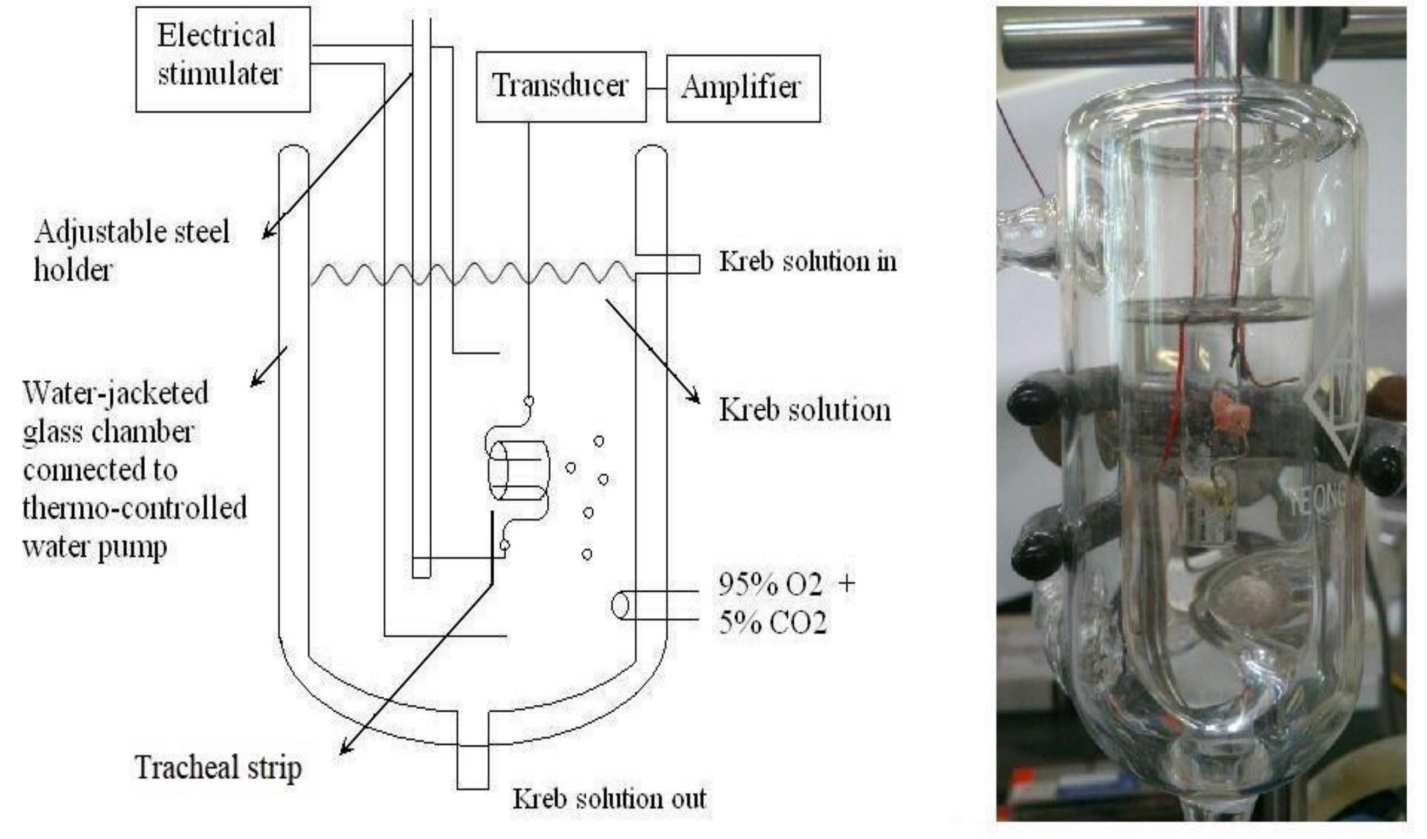
Schematic diagram (left) and actual photo (right) describing the measurement of tension in isolated rat tracheal smooth muscles.

The purest chemicals that could be found were used. By way of Ferring Pharmaceutical Taiwan Ltd., commercial desmopressin nasal spray was secured. Sigma provided all additional chemical reagents (St. Louis, MO, USA). As a tracheal contraction aid, methacholine was used. Twenty-one healthy male Sprague-Dawley rats with a body weight of less than 200 g were humanely killed by CO_2_ gas asphyxiation, and each rat had two portions of trachea (each measuring about 5 mm in length) removed. The rats were fifteen weeks old. This study had three groups. Three groups of seven rats each were used for different tests: the resting tension test (group 1), the methacholine-induced contraction test (group 2), and the electrical field stimulation test (group 3). An animal experimental review board gave its approval to this work (LAC-2022-0065). All experimental protocols were approved by Shuang Ho Hospital ethics committee of Taipei Medical University. All methods were carried out in accordance with relevant guidelines and regulations. The tracheal specimen was mounted using two steel hooks and placed, in accordance with earlier reports 9,10, in a 30 mL muscle bath at 37°C. In a nutshell, 30 mL of Krebs solution, consisting of (in mmol/L): NaCl, 118; KCl, 4.7; CaCl_2_, 2.5; MgSO_4_•7H_2_O, 1.2; KH_2_PO_4_, 1.2; NaHCO_3_, 25.0; and glucose, 10.0. A steel hook and a 3-0 silk ligature were used to secure the upper side of the tracheal strip to a Grass FT-03 force displacement transducer (AstroMed, West Warwick, RI, USA). The strip's other side was fastened to a steel hook that was joined to the bathtub (Fig 1). Using Chart V4.2 software, a passive tension of 0.3 g was applied to the strips, and any further changes in tension were constantly recorded (PowerLab, AD Instruments, Colorado Springs, CO, USA). The tracheal strip immersed in the bath solution utilized for the future trials did not constrict when basal tension was applied, according to preliminary tests. Isolated tracheas were equilibrated in the bath solution for 15 to 30 minutes prior to drug testing, and throughout this time they were continuously aerated with a mixture of 95% O_2_ and 5% CO_2_. To investigate the contraction or relaxation responses of tracheal strips, medication dosages were gradually increased. By adding a specific volume of stock solution to the tissue bath solution, all medications were given. One control strip was left untreated in each experiment.

The trachea strip was subjected to electrical field stimulation (EFS) using two wire electrodes that were parallel to the trachea strip and linked to a direct-current stimulator (5-Hz, 5-ms pulse duration, at 50 V, trains of stimulation for 5 s) (Grass S44, Quincy, MA, USA). Each stimulation period had a 2-minute break in between to give the strip time to recover from the response. The trachea was continually stimulated at a temperature of 37°C. Desmopressin assessments included the following. (1) Tracheal smooth muscle resting tension was used to assess how the medication affected a situation that mimicked a resting trachea condition. (2) Methacholine, a parasympathetic mimic, has an effect on contraction, which can be utilized to study postsynaptic events such muscle-receptor blockage, augmentation, and second messengers. (3) Desmopressin has a third effect on electrically induced contractions: electrical stimulation of this tissue triggers transmitter (acetylcholine) release from parasympathetic nerve remnants in the trachea. Electrical stimulation does not cause contraction if there is transmitter release interference. This approach made it easier to see presynaptic events.

Drug concentrations were listed as percentages of the 30 mL bath solution. Standard deviations and mean values were used to present the data (SDs). The Student t-test was used to compare variations in mean values. At P< 0.05, differences were thought to be significant.

## Results

**Figure 2:**
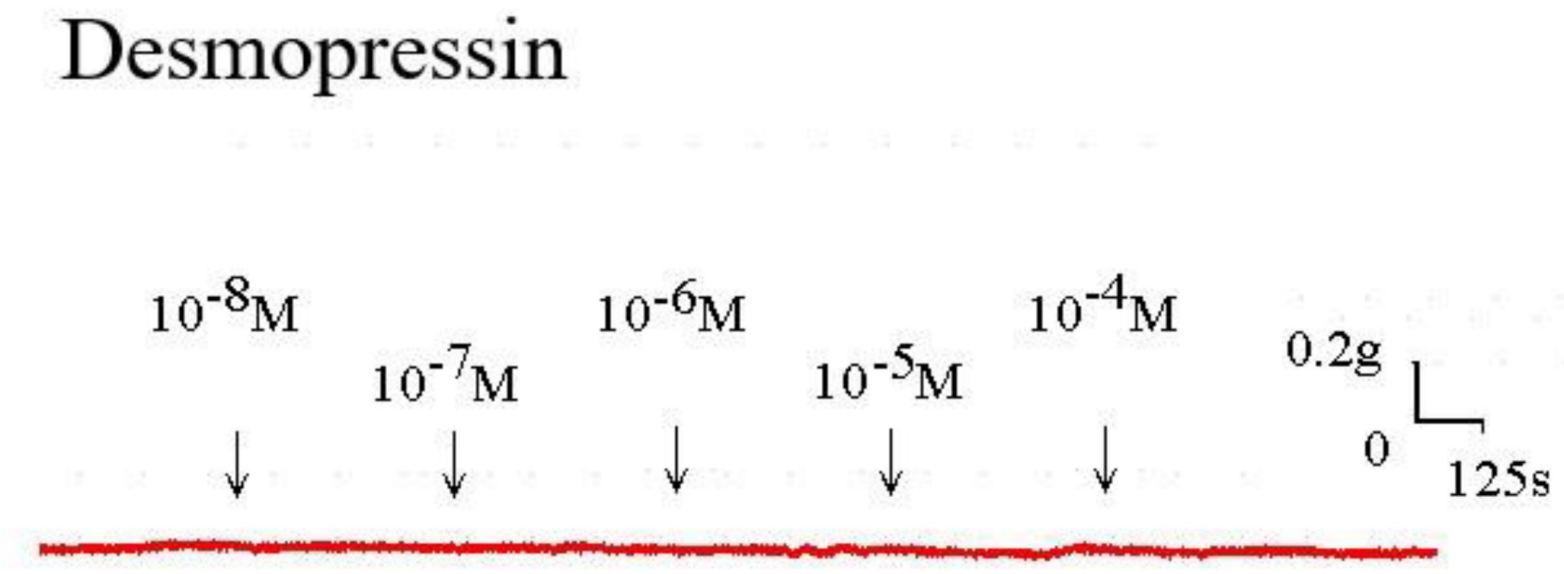
Tension changes in rat trachea after the application of various desmopressin concentrations. It alone had a minimal effect on the basal tension of trachea as the concentration increased. Original basal tension was 0.3 g. (g = gm; s = sec)

**Figure 3:**
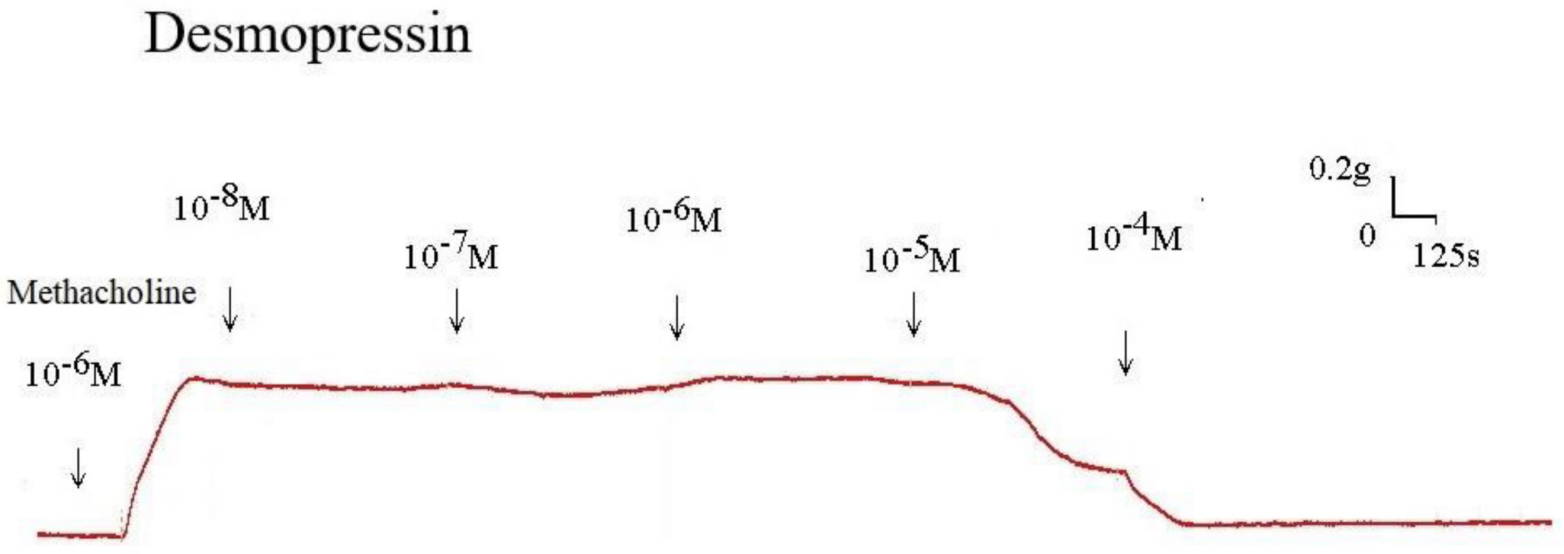
Original recording of the effects of desmopressin on 10^-6^ M methacholine-induced contraction of rat trachea. (g = gm; s = sec)

**Figure 4:**
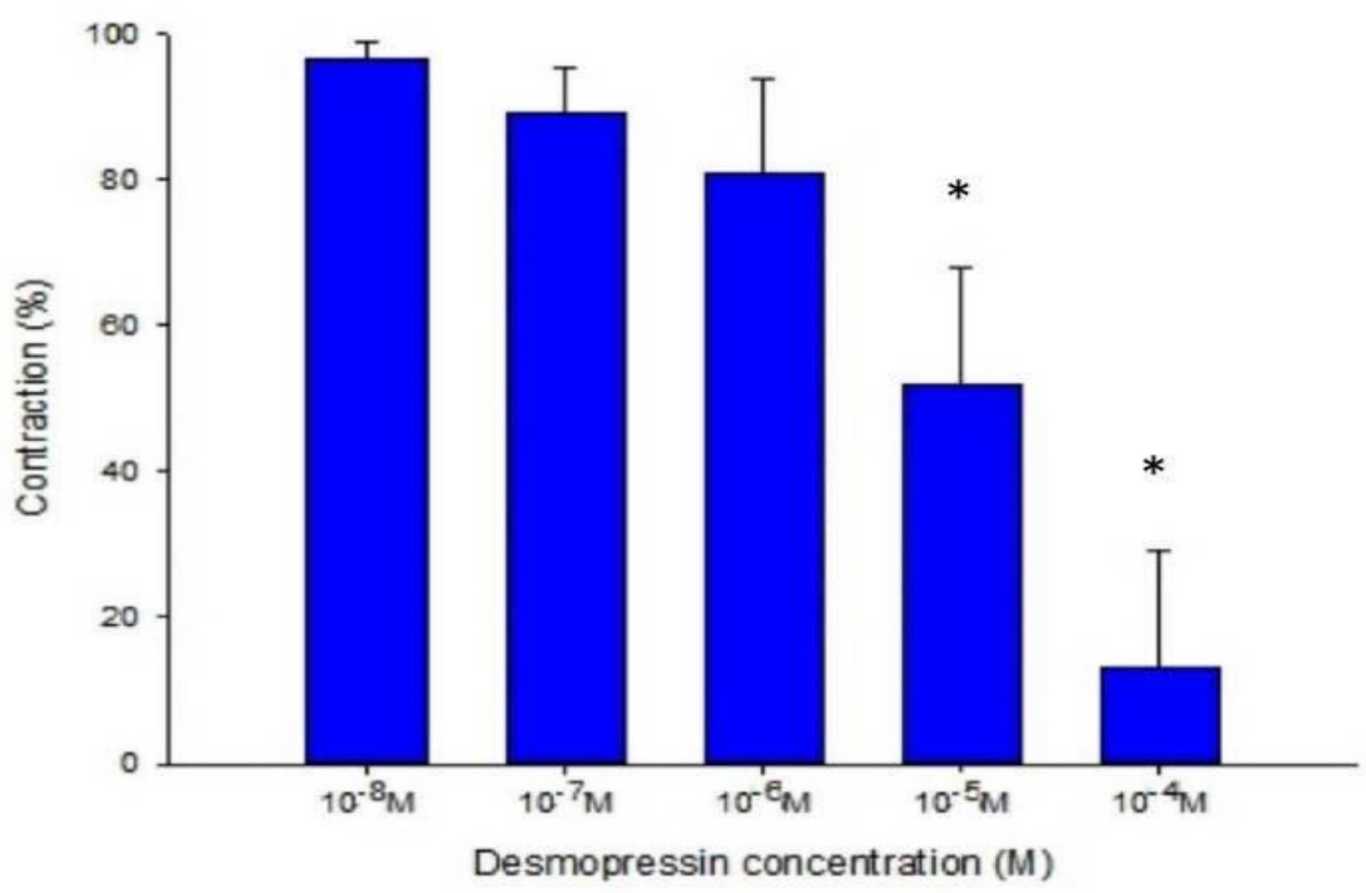
Effects of desmopressin on 10^-6^ M methacholine-induced contraction of rat trachea (contraction area calculated at 100% with no addition of desmopressin). Difference of tension between 10^-7^ M desmopressin and 10^-5^ M desmopressin or 10^-4^ M desmopressin was statistically significant (P<0.05). Results were mean ± SD (n = 7). An asterisk means statistical significance.

The strain placed on the transducer was used to estimate the contraction or relaxation of the tracheal strips. A little dosage of methacholine was all that was needed to cause tracheal constriction, and the tissue stayed constricted until the medication was rinsed off it. Desmopressin solution addition had a minimal impact on the basal tension (Fig 2). When used following the addition of a constricting drug as 10^-6^ M methacholine, it caused the trachea to relax (Fig. 3). Desmopressin had a minor effect on contraction at low doses, but it greatly relaxed the trachea smooth muscle at higher levels (Figs. 3, 4). The tension was 89.0% ± 6.4% of control values at 10^-7^ M desmopressin (Fig. 4). The tensions were lowest at 10^-5^ M and 10^-4^ M desmopressin. At 10^-5^ M and 10^-4^ M desmopressin, the tensions were 51.8% ± 16.2% and 13.0% ± 16.3%, respectively (Fig. 4). There was a statistically significant difference in tension between the specimens treated with 10^-7^ M desmopressin and those treated with 10^-5^ M or 10^-4^ M desmopressin (P < 0.05).

## Discussion

The context of the test materials should be considered when interpreting these results. The research was quick and efficient. It is crucial that this method incorporates an unbroken tracheal ring 9,10, which is far more physiologically relevant than just using smooth muscle strips. The essence of tissues and their reactions to medications give some insight, but it was difficult to pinpoint which tissue component of the trachea was responsible for drug-induced constriction. The tracheal strips employed in our study were unfinished products made of tracheal smooth muscle and cartilage. Because the other tissue components (epithelium, glands, connective tissue, nerves, and cartilage) did not contract to a sufficient degree, it appeared that the trachea's smooth muscle was the primary factor in contraction. Changes in tension were brought on by radial contraction of the tracheal ring since this technique entailed cross contraction. The contractile response seen in this investigation was likely an amalgam of the reactions of different types of muscle tissue, even though responses to medicines and electrical stimulation have been verified for comparable preparations 11-14. Meanwhile, the rats' smooth muscle and endothelium were unharmed when the isolated tracheal preparations utilized in our tests were removed. Therefore, it makes sense that the tracheal reactions to the test substances in our investigation would be analogous to those seen after spraying the trachea during an asthma episode. The impact of this medication on isolated human tracheal smooth muscle must be studied because it is extremely difficult to obtain human tissue for such investigations. Additionally, the response under *in vivo* settings may be considerably more complicated than under *in vitro* conditions. Typically, the cholinergic contracting agent used in this formulation is used in research. It is notable that the tissue relaxation brought on by desmopressin required a previous partial contraction of the smooth muscle following the application of methacholine. It should be able to evaluate the effects of common medications and potential therapeutic substances that are thought to be responsible for reducing asthma attacks. In a prior study 10, we concluded the acetylcholine (ACH) testing. Tracheal contractions are also induced by ACH. Acetylcholinesterase is responsible for catalyzing the breakdown of ACH, which prevents the contraction from continuing. Thus, ACH was not applied in this investigation. Desmopressin could reduce methacholine-induced contraction. A commercial nasal spray has 0.1 mg desmopressin acetate per mL, which could be translated into approximately 1.0 x 10^-4^ M desmopressin. Therefore, a commercial desmopressin nasal spray might reduce contraction of tracheal smooth muscle during an asthmatic attack. But the concentration effect of desmopressin in tracheal smooth muscle during a nasal spray use remains unclear and requires further studies. In addition, the mechanism by which desmopressin affected the trachea smooth muscle is unknown and requires further investigation.

**Figure 5:**
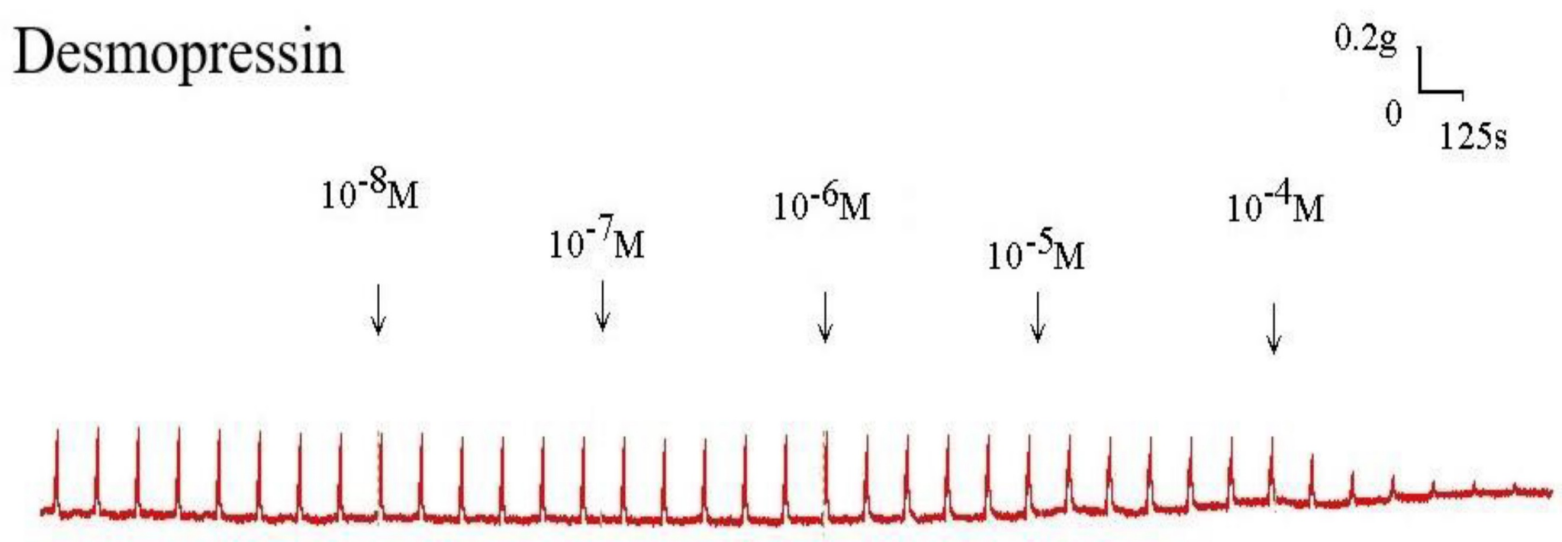
Original recording of the effects of desmopressin on electrically induced tracheal smooth muscle contractions. Higher doses of desmopressin also decreased the spike contraction induced by EFS. (g = gm; s = sec)

**Figure 6:**
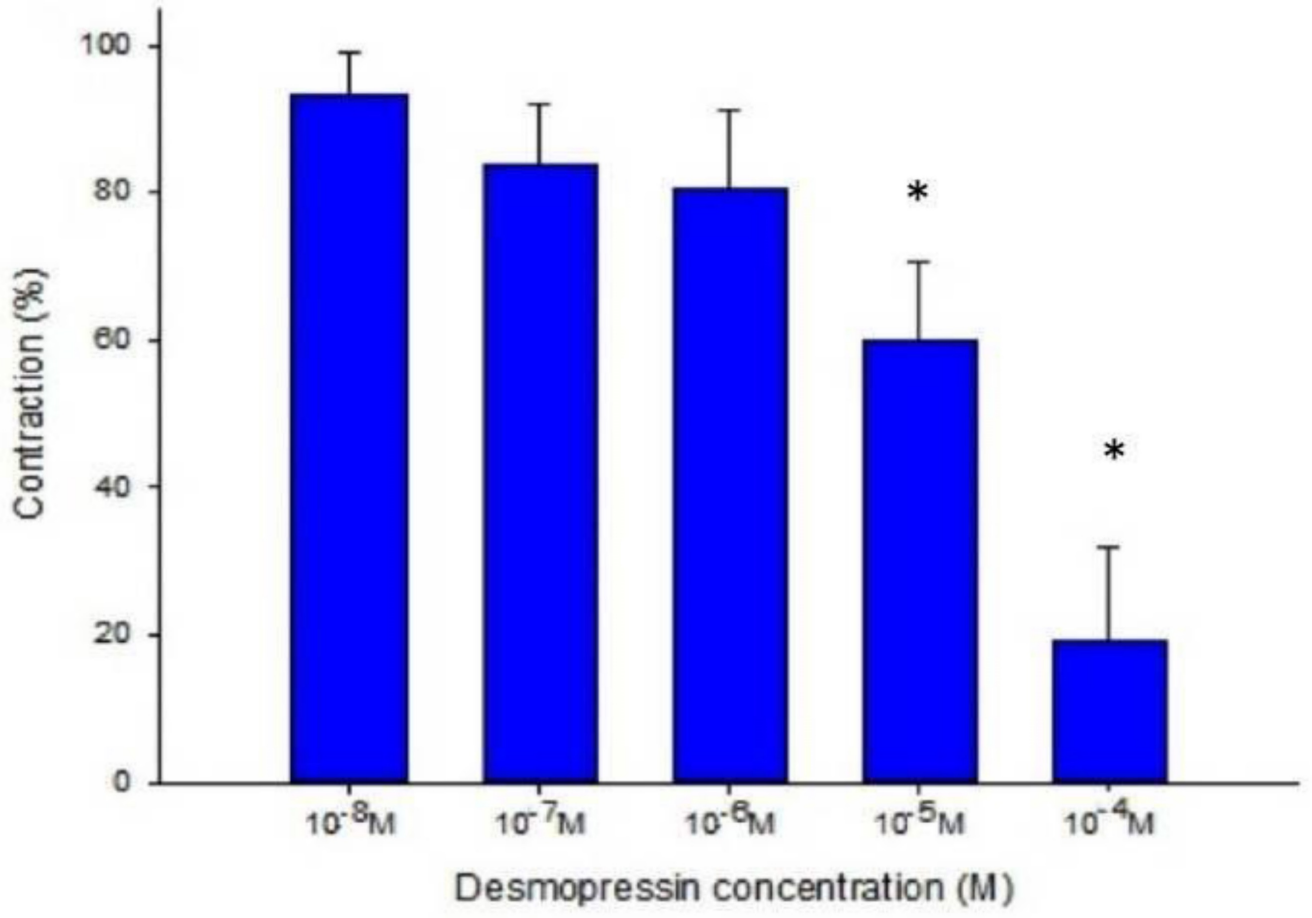
Effects of desmopressin on electrically induced tracheal smooth muscle contractions (contraction area calculated at 100% with no addition of desmopressin). Peak tension value of the tracheal strip evoked by EFS during the addition of 10^-5^ M or 10^-4^ M desmopressin was significantly lower than that at the addition of 10^-7^ M desmopressin (P<0.05). Results were mean ± SD (n = 7). An asterisk means statistical significance.

Desmopressin also prevented the EFS-induced spike constriction (Figs. 5, 6). When 10^-7^ M desmopressin was added, the tracheal strip tension peaked at 83.8% ± 7.9%, compared to peaks at 10^-5^ M and 10^-4^ M desmopressin of 59.8% ± 10.6% and 19.3% ± 12.6%, respectively (Fig. 6). When 10^-5^ M or 10^-4^ M of desmopressin were added, the peak tension value of the tracheal strip elicited by EFS was considerably lower than when 10^-7^ M of desmopressin was added (P < 0.05).

A typical experimental tool is EFS. It causes the release of endogenous neurotransmitters and stimulates nerve terminals in the tissue being examined, causing smooth muscle contraction. For instance, ipsilateral cervical sympathetic ganglionectomy eliminates EFS-induced spike contraction of canine nasal mucosa, which is thought to be caused by the contraction of nose vascular smooth muscles 15. Thus, it has been established that sympathetic innervation is a mediator of EFS-induced spike contraction of isolated canine nasal mucosa 15. In the current work, it was hypothesized that the stimulation of parasympathetic innervation caused the EFS-induced spike contraction of the tracheal smooth muscle. Thus, the EFS-induced tracheal contraction decreased as desmopressin concentration rose. These findings suggested that a desmopressin nasal spray could inhibit the parasympathetic innervation that causes the contraction of the tracheal smooth muscle. Both terbutaline and salmeterol are β_2_-adrenergic receptor agonists. In this design, they ought to exert the same influence on the isolated trachea. Our group has reported on the effects of terbutaline, a specific bronchodilator, on the isolated rat tracheal smooth muscle 16. The effects of desmopressin and terbutaline were comparable. Methacholine-induced contraction and EFS-induced spike contraction of the isolated trachea were suppressed by both drugs. Another first-line bronchodilator for asthma is corticosteroids. Prednisolone (Kidsolone) is an anti-inflammatory drug that is used for patients with chronic obstructive airway diseases (COAD), such as asthma and chronic obstructive pulmonary diseases. We have completed the testing of prednisone (kidsolone) in a previous paper 17. It is unclear, though, if the advantages of glucocorticoids in COAD stem solely from their ability to reduce inflammation or from their ability to act through the intracellular glucocorticoid receptor and the genomic pathway. It is well known that the genetic impact of glucocorticoids happens hours or even days after exposure. There were no immediate (non-genomic) effects of steroids on tracheal smooth muscle in this *in vitro* study. The responses were obviously different between desmopressin and corticosteroids. Additionally, basal tension had very little of an impact on desmopressin concentrations. Desmopressin decreases arterial blood pressure in the anesthetized rat and relaxes isolated segments of aorta and pulmonary artery 18-20. Arginine vasopressin can interact with at least two types of receptors, V1 and V2 receptors. Vasopressin V1 receptors mediate the vasoconstriction elicited by this peptide and vasopressin V2 receptors may mediate vasodilatation in some vascular beds. The modulator role of nitric oxide and prostaglandins in the vascular actions of arginine vasopressin has been suggested 21. Desmopressin exerts powerful endothelium-dependent relaxation of human renal arteries, probably through stimulation of V2-like receptors that may bring about the release of dilating prostaglandins 22. Desmopressin has been shown in prior research 23 to increase the generation of nitric oxide in human lung microvascular endothelial cells, which may be due to the activation of V2-like receptors. It obviously had the dilatation effects on trachea in this study. The findings of this study are quite intriguing. To further understand these events, more research is required.

Desmopressin can be administered orally in a thrice-daily regimen. This study demonstrates it is a bronchodilator, though its precise mechanism of action for the treatment of chronic asthma remains to be established. The results of this study showed that high concentrations of desmopressin might inhibit parasympathetic function of the trachea, and it could also reduce methacholine-induced contraction. Accordingly, in addition to nocturia symptoms, it could effectively treat an asthmatic attack. The response could be far more difficult in the *in vivo* setting than it was in the *in vitro* setting. What will be the blood pressure at those doses, for example, in terms of desmopressin safety? How about the retention of water? Is the potency of desmopressin greater than that of inhaled corticosteroids? Are there any safe human doses that correspond to such levels? Is it dangerous to administer such a drug at such dosages to those elderly patients? Both oral and intranasal forms of desmopressin are generally well tolerated during treatment, with little adverse effects. If desmopressin is prescribed, the small risk of hyponatremia can be reduced by adhering to the guidelines for indications, dose, and safety precautions 24. However, further research is required before desmopressin is used to treat asthma. The Global Initiative for Asthma (GINA) Science Committee's 2024 update states that bronchodilators and corticosteroids remain the initial lines of treatment for asthma. There is no information on using desmopressin to treat asthma. In this investigation, desmopressin demonstrated obvious dilatation effects on the trachea. The findings of this study motivate additional research to shed more light on these events.

## Conclusion

According to this study, desmopressin at high quantities might prevent the trachea's parasympathetic activity. When given to an aging patient with nocturia, desmopressin nasal spray might lessen asthmatic attacks because it could decrease parasympathetic function and lessen the contraction of tracheal smooth muscle brought on by methacholine.

## Ethical approval

An animal experimental review board gave its approval to this work (LAC-2022-0065). All experimental protocols were approved by Shuang Ho Hospital ethics committee of Taipei Medical University.
